# Functional Surfaces in Biology; Springer: Berlin, Germany, 2009; 2 Vols.; Edited by Stanislav N. Gorb

**DOI:** 10.3390/biomimetics2020005

**Published:** 2017-04-11

**Authors:** Werner Nachtigall

**Affiliations:** Department of Zoology, University of Saarland, Gebäude 6, 66041 Saarbrücken, Germany; nachtigall.werner@t-online.de

The editor of these book, Stanislav N. Gorb, is one of the most well-known experts in his field—especially in biological adhesion phenomena and the function of biological surfaces in general. He has conducted intensive research at various scientific institutions, as well as technical institutions such as the Max-Planck Institute for Metal Research in Stuttgart. Now, he is Professor of the Department of Functional Morphology and Biomechanics and Director at the Zoological Institute of the University of Kiel. His research is of high interest, especially for entomologists. Many of his investigations were carried out on the insect tarsus; they deal with known fundamental phenomena from insects, such as adhesion and anti-adhesion on biological and non-biological surfaces. Both of his books are written in English and have been carefully edited [1,2]. They present results not only of Gorb’s own research but also of other working groups. Totalling 672 pages, the books represent a rich source of information, comprehending 30 detailed reports written by 46 authors and co-authors. Here, the reader can find almost everything regarding current knowledge in this research field.

Volume 1 consists of seven topics: (1.1) *Protection and Defence*; (1.2) *Anti-Wetting*; (1.3) *Transport*; (1.4) *Aerodynamics*; (1.5) *Acoustics*; (1.6) *Sensory Systems*; and (1.7) *Optics* [1]. Volume 2 contains two parts: (2.1) *Adhesion Enhancement* and (2.2) *Adhesion Reduction* [2]. The single chapters within these topics are shortly discussed below.

(1.1) *Protection and Defence*: In this part, protective seed slimes and defence properties of the integument of tenthredinid larvae are discussed. (1.2) *Anti-Wetting*: Chapters on the aspects of water repellence in geckos, spiders, leafhoppers, representatives of Heteroptera, and of the water fern *Salvinia* form another large part of the first Volume. (1.3) *Transport*: Somewhat off topic is the work on “inner surfaces” dealing with the plant water transport. (1.4) *Aerodynamics*: This part contains a study on the origin and evolution of feathers. The heading “*Aerodynamics*” does not really reflect the content here, because the work is not about fluid mechanical effects. (1.5) *Acoustics*: This work addresses specialised structures of the sound emission organs in *Urania* moths. (1.6) *Sensory Systems*: The impressive work on sensory hairs in the crickets *Achaeta* and *Gryllus* is not strongly related to the topic of surfaces. (1.7) *Optics*: The four studies dealing with optics present research on the wing-scales of Lepidoptera, aspects of the microstructures causing reflections, surface colours of insects, and biophotonics in general. (2.1) *Adhesion Enhancement*: This contains five contributions and represents the most comprehensive part of the book. This topic includes chapters on the structure and properties of the Echinoderm tube foot, settlement of barnacles, insect arolium with special reference to Hymenoptera, attachment devices of some mountain-stream fish and geckos. (2.2) Especially interesting are the mechanisms of *Adhesion Reduction* on the surfaces of the carnivorous pitcher plant *Nepenthes*.

The contributions are in the form of specific research reports and reviews. Many of the latter contain very detailed and extremely helpful reference lists. In my opinion, the selection and composition of most of the chapters are suitable. Rare exceptions might be Part (1.4) and Part (1.6). Most of all, I enjoyed the following four studies: The work of Cerman, Striffler and Barthlott (in Part 1.2) might be the most exciting one from the biomimetic point of view, because it may result in a new energy saving mechanism by reducing the skin friction of ship hulls. I have never read such a good review on the origin of birds’ feathers as that written by Alibardi (Part 1.4). From Ingram’s work (in Part 1.7), I learned a lot about the actual aspects of photonics in butterflies. Moreover, I enjoyed rediscovering a number of Gorb’s fundamental research results in the single chapters to which he contributed as co-author.
